# C1632 suppresses the migration and proliferation of non‐small‐cell lung cancer cells involving LIN28 and FGFR1 pathway

**DOI:** 10.1111/jcmm.17094

**Published:** 2021-12-16

**Authors:** Jing‐yi Chen, Yu‐jing Chen, Lu Liu, Xiang‐xiang Jin, Zhe Shen, Wen‐bin Chen, Teng Yang, Si‐bei Xu, Guang‐bao Wang, Yi‐nuo Cheng, De‐zhi Cheng, Zhi‐guo Liu, Xiao‐hui Zheng

**Affiliations:** ^1^ Chemical Biology Research Center School of Pharmaceutical Sciences Wenzhou Medical University Wenzhou Zhejiang China; ^2^ Department of Thoracic Surgery The First Affiliated Hospital of Wenzhou Medical University Wenzhou Zhejiang China

**Keywords:** anti‐migration, anti‐proliferation, chemotherapy resistance, FGFR1, LIN28, non‐small cell lung cancer

## Abstract

Chemoresistance and migration represent major obstacles in the therapy of non‐small‐cell lung cancer (NSCLC), which accounts for approximately 85% of lung cancer patients in clinic. In the present study, we report that the compound C1632 is preferentially distributed in the lung after oral administration in vivo with high bioavailability and limited inhibitory effects on CYP450 isoenzymes. We found that C1632 could simultaneously inhibit the expression of LIN28 and block FGFR1 signalling transduction in NSCLC A549 and A549R cells, resulting in significant decreases in the phosphorylation of focal adhesion kinase and the expression of matrix metalloproteinase‐9. Consequently, C1632 effectively inhibited the migration and invasion of A549 and A549R cells. Meanwhile, C1632 significantly suppressed the cell viability and the colony formation of A549 and A549R cells by inhibiting DNA replication and inducing G0/G1 cell cycle arrest. Interestingly, compared with A549 cells, C1632 possesses the same or even better anti‐migration and anti‐proliferation effects on A549R cells, regardless of drug resistance. In addition, C1632 also displayed the capacity to inhibit the growth of A549R xenograft tumours in mice. Altogether, these findings reveal the potential of C1632 as a promising anti‐NSCLC agent, especially for chemotherapy‐resistant NSCLC treatment.

## 1 INTRODUCTION

Lung cancer is one of the most common malignant tumours and is responsible for 25% of cancer‐related deaths each year.^1^, ^2^ Approximately, 85% of lung cancer patients have been clinical diagnosed as non‐small cell lung cancer (NSCLC); thus, the treatment of NSCLC has been an urgent health issue worldwide.^3^ Progress in this area has been substantial and promising over the past 20 years with the advent of various targeted therapies^4^ and immunotherapy^5^ in some advanced NSCLC patients.^6^ For instance, the use of small molecule tyrosine kinase inhibitors, such as EGFR tyrosine kinase inhibitor,^7^, ^8^, ^9^, ^10^, ^11^ ALK inhibitors^12^, ^13^ and ROS1 inhibitors,^14^ has achieved unprecedented survival benefits in some selected patients. However, small molecule tyrosine kinase inhibitors could only be used for a small minority of NSCLC patients with gene alterations.^15^ Consequently, the overall cure and survival rates of NSCLC remain low.^1^, ^16^ Thus, continued research into new small molecule inhibitors that significantly suppress NSCLC cell motility and invasiveness as well as proliferation is desired.

LIN28, which is an RNA‐binding protein consisting of LIN28A and LIN28B,^17^ is an important regulator of miRNAs and mRNAs.^18^, ^19^ LIN28 regulates not only the translation of mRNAs that play a key role in cell growth and metabolism but also the biogenesis of miRNAs.^20^, ^21^ Recently, studies have found that LIN28 levels are increased in clinical NSCLC tissues, and this is correlated with the increased ability of cell migration and proliferation,^22^, ^23^, ^24^ and drug resistant of human lung cancer cells.^25^ Thus, LIN28 is an appealing therapy target for small molecule drugs in the NSCLC treatment field.

Fibroblast growth factor receptor 1 (FGFR1), an oncogenic receptor tyrosine kinase, plays fundamental roles in stimulating cell proliferation and migration.^26^ Importantly, FGFR1 expression is found to be substantially increased in clinical NSCLC tissues compared with the adjacent peritumoural tissues.^27^, ^28^ Meanwhile, several FGFR1 inhibitors are under early‐phase clinical trials of NSCLC treatment.^29^, ^30^, ^31^ For instance, AZD4547 (AstraZeneca, NCT01213160) and BGJ398 (Novartis; NCT01004224; NCT01697605) possessed potential benefits in advanced NSCLC patients.^32^, ^33^, ^34^ All these studies suggested that FGFR1 inhibition could serve as an important therapeutic target in NSCLC.^27^ Unfortunately, FGFR1‐targeted therapies would result in drug resistance which finally led to the failure of targeted therapies.^1^, ^26^, ^28^ Thus, it is reasonably speculated that multitargeted small molecule inhibitors that inhibit FGFR1 along with other functional proteins, such as LIN28, may exert the strongest effects in the clinical treatment of NSCLC.

The anxiolytic compound C1632,^35^, ^36^ has been demonstrated to have LIN28 inhibitory effect in our previous work.^37^ In the present study, we discovered that C1632 mainly accumulates in lungs of mice after oral administration with high bioavailability. Furthermore, C1632 suppressed the expression of LIN28 as well as the phosphorylation of FGFR1 in a dose‐dependent manner in NSCLC A549 and A549R cells. We also investigated the possibility that C1632 acts as a small molecule anticancer drug targeting both LIN28 and FGFR1 in the treatment of NSCLC, especially for cisplatin‐resistant NSCLC.

## 2 MATERIALS AND METHODS

### 2.1 Cell culture and reagents

The human normal foetal lung fibroblast cell MRC5, NSCLC cell lines A549, and cisplatin‐resistant NSCLC cell lines A549R were obtained from Chinese Typical Culture Center from 2014 to 2015. MRC5 was cultured in Dulbecco's modified Eagle's medium (DMEM, Gibco), which was supplemented with 15% foetal bovine serum (Gibco) and 1% penicillin/streptomycin. A549 and A549R cell lines were maintained in Roswell Park Memorial Institute (RPMI) 1640 (Gibco) that was supplemented with 10% foetal bovine serum (Gibco) and 1% penicillin/streptomycin. All cells were cultivated at 37°C under 5% CO_2_.

C1632, with the purity of 99.86%, was synthesized in Chemical Biology Research Center, Wenzhou Medical University as previously described.^37^ Ponatinib (Item No. 1239124) was purchased from J&K Scientific.

### 2.2 Western blot assay

The total cell protein was extracted and denaturized at 95°C for 10 min. Samples were separated in SDS‐PAGE gel, then transferred to a PVDF membrane. Then this membrane was blocked with 5% non‐fat milk (solved in TBST) for 2.5 h at room temperature. Blots were probed with relevant primary antibodies against FGFR1 (CST), p‐FGFR1 (CST), MAPK (CST), p‐MAPK (CST), FAK (CST), p‐FAK (CST), MMP‐9 (CST), LIN28B (CST), GAPDH (CST), and β‐actin (proteintech) overnight at 4°C, respectively. Then the lanes were detected with HRP‐conjugated secondary antibodies and visualized using WESTAR SUPERNOVA kit (CYANAGEN).

### 2.3 Cell adhesion assay

The cell adhesion assay was performed as indicated previously.^38^ Briefly, the 96‐well plate was coated with human fibronectin (Millipore), and then cells were seeded into the 96‐well plate and cultured for another 1 h at 37°C in a 5% CO_2_ incubator. Finally, rinsing the plate before fixed with 10% formalin and stained with crystal violet. As followed, wash the plate with ddH_2_O and add 100 μl acetic acid (33%) into the plate to dissolve the crystal violet. The absorbance at 560 nm was detected by Synergy H1 Multi‐Mode Reader (BioTek). Relative number of cells attaching to extracellular matrix was evaluated using the following equation: mean OD of treated cells/mean OD of control cells.

### 2.4 Transwell assay

Transwell assay was carried out according to the manufacturer's instructions provided by Transwell Kit (Corning Costar). Briefly, cells were pretreated with indicated concentrations of C1632 for 5 days, then harvested and re‐seeded into insert (transwell permeable support) containing 100 μl serum‐free DMEM medium. The insert was placed into 24‐well plate containing 600 μl of DMEM medium extra added with 10% FBS. 24 h later, cells on the upper surface of the insert were removed with cotton‐tipped swabs. And cells on backside surface of the insert were fixed with 10% formalin, then stained with crystal violet. The insert was washed three times with ddH_2_O before it is subjected to Nikon Ti microscope observation. Additionally, these inserts were dissolved in 500 μl acetic acid (33%) separately, and the absorbance at 560 nm was detected by the spectrophotometer (DTX880, Beckman Coulter).

### 2.5 Scratch‐wound assay

The cells scratch‐wound assay was performed as previous reported.^38^ The cells were seeded in a 6‐well plate and then cultured in DMEM medium containing indicated concentration of C1632 or 0.01% DMSO for 5 days. A denuded area was created across the diameter of dish by a yellow tip as the cell density up to 95%. Then cells were maintained in a serum‐free medium throughout the test. Phase‐contrast images were taken at the indicate time by Nikon Ti microscope and analysed with Axiovision Rel.4.8 software.

### 2.6 Immunofluorescence assay

Performing the immunofluorescence (IF) assay as previously described.^39^ Briefly, cells were seeded to slide, fixed with 4% paraformaldehyde, and permeabilized using 0.5% Triton X‐100. Next, cells were incubated with primary antibodies against FAK (CST), followed by incubating with secondary antibodies (DyLight 488‐conjugated anti‐mouse). DAPI was used to stain the nuclear. The fluorescence image was captured by using Nikon Ti microscope and the quantitative analysis was carried out by ImageJ.

### 2.7 3‐(4,5‐dimethylthiazol‐2‐yl)‐2,5‐diphenyltetrazolium bromide assay

The cell viability of MRC5, A549, and A549R were assessed by the 3‐(4,5‐dimethylthiazol‐2‐yl)‐2,5‐diphenyltetrazolium bromide (MTT) assay.^39^ Cells were treated with indicated concentration of C1632 or 0.01% DMSO (Control) for 48 h before 20 μl/well MTT (0.5 mg/ml) was added for another 4 h. The reaction product formazan was dissolved in 100 μl DMSO and the absorbance at 560 nm was determined by the spectrophotometer (DTX880, Beckman Coulter).

### 2.8 Edu staining assay

Edu staining assay was carried out according to Edu staining Kit (Beyotime). First, A549 or A549R cells were seeded in 6‐well plates and cultured in RPMI medium 1640 containing 10% FBS, then treated with C1632 (15, 30, or 60 mg/L) for 5 days. Subsequently, cells were incubated with Edu for 3 h, fixed with 4% paraformaldehyde for 15 min, and permeated with 0.3% Triton X‐100 for another 15 min. Then cells were incubated with the Click Reaction Mixture provided by Edu staining Kit for 30 min at room temperature in dark and then stained with DAPI. Finally, the stained cells were scanned and imaged under Nikon Ti microscope.

### 2.9 Colony cloning assay

First, cells were treated with indicated concentrations of C1632 or 0.01% DMSO for 5 days. Then these cells were separated into single cells that were directly used for cloning. During the process of cloning, C1632‐treated A549 or A549R cells were still maintained in DMEM plus 10% FBS medium containing the indicated concentration of C1632 (15, 30, and 60 mg/L), while the control group was cultured with 0.01% DMSO. Both culture media were changed for every 2 days until 10 days. The number of forming colonies in C1632 or 0.01% DMSO‐treated groups was counted and the images were taken.

### 2.10 Cell cycle distribution analysis

Cells were cultured in the absence or presence of 15, 30, and 60 mg/L of C1632 or 0.01% DMSO for 5 days, trypsinized, washed, and stained with propidium iodide before cell cycle distribution was assessed on a flow cytometer (BD FACSCalibur, BD Biosciences).

### 2.11 Annexin V/PI apoptosis assay

Cells were seeded in 6 cm^2^ dish with a density of 3.0 × 10^5^ cells per dish and treated with C1632 with final concentration of 15, 30, and 60 mg/L or 0.01% DMSO for 5 days. Then the assay was performed following the protocol provided by the Annexin V/PI Apoptosis Kit (Sigma) and was assessed on a flow cytometer (BD FACSCalibur, BD Biosciences).

### 2.12 Senescence‐associated β‐galactosidase (SA‐β gal) activity assay

The assay was set up following the β‐galactosidase (SA‐β gal) staining Kit, which was obtained from Sigma. In brief, cells were washed once with PBS and fixed with stationary liquid provided in the kit at room temperature for 15 min. Next, the cells were incubated overnight at 37°C in dark with the 1 ml working solution containing 0.05 mg/ml 5‐bromo‐4‐chloro‐3‐indolyl‐b‐d‐galactopyranoside (X‐gal) and observed under a normal light microscope (Nikon).

### 2.13 RNA extraction and real‐time quantitative RT‐PCR

Total RNA was extracted using RNAiso Plus (Takara) according to the manufacturer's instructions. cDNA was synthesized using PrimeScript II first‐strand cDNA Synthesis Kit (Takara), followed by amplification with RealStar Power SYBR Mixture (GenStar). miRNAs were quantified using the stem‐loop method, and U6 snRNA was adopted as internal control. Specific primer sequences were designed as previously reported.^40^ qPCR was carried out with a LightCycler 480 Real‐Time PCR system (Roche). Data were analysed using the comparative Ct (2−ΔΔCt) method.^41^ All experiments were performed in three independent experiments. qPCR primers for mRNA detection were as follows: GAPDH‐forward 5′‐CCCATGTTCGTCATGGGTGT‐3′; GAPDH‐reverse 5′‐TGGTCATGAGTCCTTCCACGATA‐3′; LIN28A‐forward 5′‐CAACCAGCAGTTTGCAGGTGG‐3′; LIN28A‐reverse 5′‐GCGGTCATGGACA GGAAGCC‐3′; LIN28B‐forward 5′‐ATATTCCAGTCGATGTATTTGTACA‐3′; LIN28B‐reverse 5′‐TGGGTCTTCTTTCACTTCCTAA‐3′.

### 2.14 Animal studies

The male Sprague‐Dawley (SD) rats weighing 250 ± 20 g, 4‐week‐old female BALB/c‐nu mice were obtained from the Animal Center of Wenzhou Medical University. Rats were kept under standard laboratory conditions with food and tap water available ad libitum. All experimental procedures and protocols were reviewed and approved by the Animal Care and Use Committee of Wenzhou Medical university and were in accordance with the Guide for the Care and Use of Laboratory Animals.

### 2.15 Development of UHPLC‐MS/MS method for determining C1632

Agilent 1290 UHPLC system and 6420 series Triple‐Quadrupole Tandem Mass Spectrometer (Agilent Corporation) maintained at 35°C with a ZORBAX Eclipse Plus C18 column (1.8 μm, 2.1 × 50 mm). The mobile phase was a gradient elution program consisting of solvent A with solvent B at a flow rate of 0.4 ml/min. Mobile phase A was 0.1% formic acid in water (v/v), and mobile phase B was acetonitrile. The optimal gradient elution program was as follows: 0–0.5 min, linear from 80% to 5% A; 0.5–1.5 min, 5% A; 1.5–1.6 min, linear from 5% to 80% A. The post‐time was 1.3 min for equilibration of the column and the total runtime was 1.8 min. The mass spectrometer was acquired in an ESI‐positive mode with multiple reaction monitoring (MRM). Instrument control and data acquisition were performed on the MassHunter Agilent Software (version B.07.00).

The desolation gas (nitrogen) flow was set to 10 L/h: the capillary voltage: 4000 V; nebulizing gas and drying gas (both nitrogen): 45 psi and 350°C, respectively. The MRM mode with m/z 282.2→76.9 for C1632 and m/z 282.1→194.0 for IS was used for quantitative analysis.

### 2.16 Pharmacokinetic study

Male SD rats were starved for 12 h before the experiment. But, tap water was available ad libitum. C1632 was administered orally or intravenously at the dose of 20 mg/kg (p.o.), 4 mg/kg (i.v.), respectively (*n* = 6). Blood samples were collected into heparinized polythene tubes 0.083, 0.25, 0.5, 1, 2, 4, 6, 9, 12, 24 h after dosing. As followed, the samples were centrifuged at 14,954 *g* for 10 min. Add acetonitrile (400 µl) and IS (20 µl) into collected plasma samples (100 µl). Thereafter, the samples were vortexed for 2 min, followed by centrifugation at 14,954 *g* for 10 min. Remove the supernatants to 1.5 ml tube and the sample is ready for detection by established UHPLC‐MS/MS assay. The injection volume is 6 µl. The pharmacokinetic parameters were determined using DAS software (Version 3.0).

### 2.17 Tissue distribution study

Twenty‐four mice were randomly divided into four groups (six mice for each group, one group for each time point) and received 20 mg/kg (i.v.) of C1632 by oral administration. The mice were euthanized by decapitation at 0 (blank group), 0.25, 2 and 6 h after C1632 was given. Tissues were collected and washed with normal saline, then homogenized and subjected to sample preparation. Subsequently, the concentration of C1632 was determined by UHPLC‐MS/MS.

### 2.18 Microsomal incubation assay

The microsomal incubation assay^41^ was performed at 37°C in a 200 μl incubation system, which consisted of 3.4 mg/ml pooled rat liver microsomes, 100 μM C1632, and probe substrates (5 μM CYP3A2‐midazolam; 10 μM CYP2D1‐dextromethorphan; 250 μM CYP2C11‐tolbutamide; 10 μM CYP2B1‐bupropion; and 0.1 M Tris–HCl [pH 7.4]). After 5 min of incubation, 1 mM NADPH was added, and the assay was terminated after 30 min by cooling at −80°C. Next, 0.2 ml acetonitrile and 20 μl IS (100 ng/ml) were added to the reactant. Finally, the solution was thoroughly vortexed and centrifuged at 12,000 rpm for 10 min. The supernatant was analysed by UHPLC‐MS/MS.^42^

### 2.19 NSCLC A549R Xenograft Models construction

A total of 1.0 × 10^6^ A549R cells were inoculated subcutaneously into the dorsal flank of the nude mice in 100 µl phosphate‐buffered saline (PBS). Mice were randomly divided into control and C1632 treatment groups (*n* = 4 per group). Mice were i.p. injected every other day for 18 days (A549R) at a dose of 30 mg/kg in 100 µl PBS per mouse. The tumours were resected, and their size was estimated from the measurements of weight or the longest diameter across the tumour and the corresponding perpendicular diameter.

### 2.20 Knockdown of LIN28B by siRNA

LIN28B was knocked down in A549 and A549R cells by siRNA. siRNA sequences were siRNA_1‐UGCGCAUGGGAUUUGGAUU and siRNA_2AGGGAAGACACUACAGAAA; siRNAs were transfected into cells using Lipofectamine RNAiMAX (Life Technologies) following manufacturer's instructions. SiRNA with scramble sequence was used as a negative control. Cells were assayed 72 h after transfection.

### 2.21 Statistical analysis

Statistical analyses were performed with GraphPad Prism 5 using a two‐tailed Student *t*‐test or two‐way ANOVA with the *p* values. Values are presented as mean ± standard deviation (SD). *p* values < 0.05 were considered statistically significant (*p < 0.05; **p < 0.01; ***p < 0.001).

## 3 RESULTS

### 3.1 C1632 is preferentially distributed in the lung after oral administration in vivo with high bioavailability and limited inhibitory effects on CYP450 isoenzymes

**FIGURE 1:**
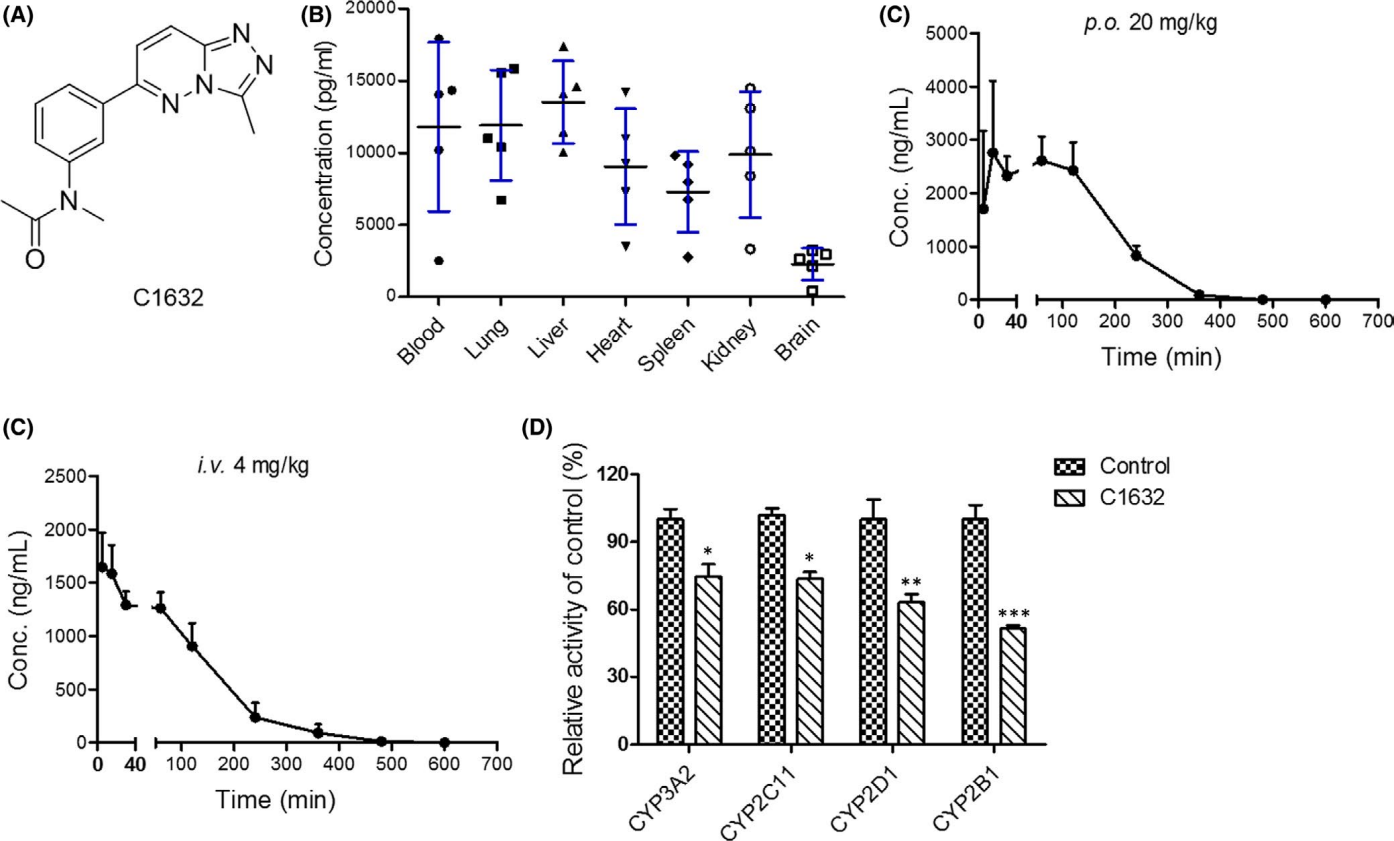
C1632 is preferentially distributed in the lung after oral administration in vivo with high bioavailability and affects the activity of CYP450 isoenzymes. (A) Chemical structure of C1632. (B) Mean concentration of C1632 in tissues at 0.25 h after tail vein administration of 20 mg/kg C1632 in the mouse. (C and D) Bioavailability of C1632. The mice were orally (C) or intravenously (D) administered with C1632 at a dose of 20 mg/kg or 4 mg/kg, respectively. Blood samples were collected at the indicated time points. UHPLC‐MS/MS was employed to determine the concentration of C1632. The concentration–time curve was plotted (*n* = 6). € C1632 inhibits the enzymatic activity of various CYP450s. The microsomal incubation assay was employed to study the inhibitory effects of C1632 on CYP3A2, CYP2B1, CYP2C11, and CYP2D1 of the rat. The probe substrates were added into the system, and their metabolites were detected. Relative enzymatic activity was calculated and plotted. Values are the average ± SD of three independent experiments. *p* values were calculated using the unpaired Student's *t*‐test (*p < 0.05, **p < 0.01, ***p < 0.001)

The concentration–time curves of C1632 (Figure 1A) in mouse plasma, heart, liver, spleen, lung, kidney, and brain after oral administration (20 mg/kg) are shown in Figure S1. The tissue distribution results indicated that C1632 diffuses rapidly and widely into major organs, with a peak at 0.25 h (Figure S1). The level of C1632 was highest in the lung and liver, followed by the kidney, heart, and spleen (Figure 1B). As expected, the highest accumulation took place in the liver, where the majority of medicine metabolism takes place. The level of C1632 in the brain remained low, suggesting that C1632 may be effectively prevented from crossing the blood–brain barrier. The fact that the accumulation in the lung was equal to that in the liver indicates that C1632 has potential application in lung cancer therapy.

To determine the bioavailability of C1632 in rats, UHPLC‐MS/MS was applied. C1632 was delivered orally and intravenously, and blood samples were collected from the tail at indicated time points. As shown in Figure 1C, there was a second peak at 1 h in the oral administration group. So, C1632 might be distributed to the liver and intestines. The mean concentration–time curves are shown in Figure 3C,D. The pharmacokinetic parameters were calculated and are presented in Table S1. The bioavailability of C1632 is 44.45%.

In addition, a cocktail assay was performed to evaluate the impact of C1632 on CYP450s. As shown in Figure 1E, the activities of four primary CYP450 isoenzymes, CYP3A2, CYP2C11, CYP2D1, and CYP2B1, were measured in the presence or absence of C1632. Although the results indicated that C1632 could significantly inhibit their activity, the concentration of C1632 was relatively high. Therefore, the effect of C1632 on liver drug enzymes is limited.

### 3.2 C1632 suppresses the expression of LIN28 and blocks FGFR1‐mediated signalling in NSCLC A549 and A549R cells

**FIGURE 2:**
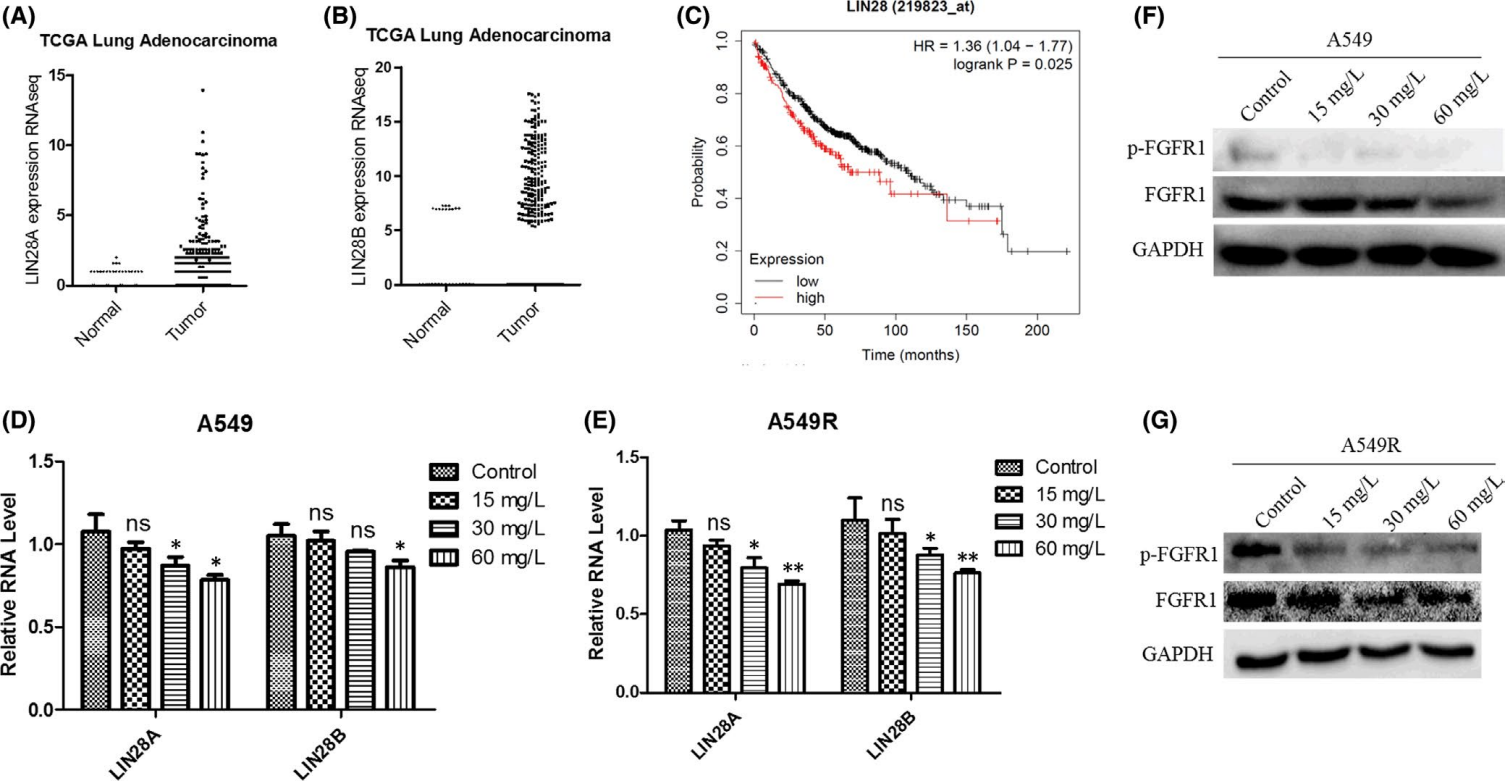
C1632 inhibits the expression of LIN28, the phosphorylation of FGFR1, and its downstream pathways in NSCLC A549 and A549R cells. (A) Plots of the expression values of LIN28A in lung adenocarcinoma cancer samples compared with normal tissues (*n* = 575). (B) The same as in A for LIN28B (*n* = 524). C Kaplane‐Meier Plotter showed the association between LIN28 expression and overall survival of lung adenocarcinoma cancer patients. Data were acquired from The Cancer Genome Atlas (TCGA). (D) A549 cells were treated with 0.01% DMSO or C1632 (15, 30, and 60 mg/L) for 5 days. Then LIN28A and LIN28B RNA was isolated for qPCR. Values are the average ± SD of three independent experiments. *p* values were calculated using the unpaired Student's *t* test (***p < 0.001). (E) The same as in C for A549R cells. (F) C1632 dose‐dependently inhibited the activation of FGFR1 and downstream proteins in A549 cells. A549 cells were treated with C1632 at indicated concentrations for 5 days before cell lysis. Western blot analysis was used to determine the protein expression of p‐FGFR and FGFR1. GAPDH was used as loading control. The results shown are representative of three replicated experiments. (G) The same as in E for A549R cells

On the basis of data from the TCGA database,^43^ we validated that both adenocarcinoma and squamous cell carcinoma of lung patients, which comprise 40% and 25% of NSCLC,^1^ respectively, express much higher LIN28 mRNA levels in tumour tissue than in normal tissue (Figure 2A,B, Figure S2). Meanwhile, survival analysis showed that the patients expressing higher LIN28 showed lower survival rates compared with patients with low LIN28 expression (Figure 2C). Additionally, blockage of the FGFR1 signalling pathway has been verified as a practical therapeutic strategy in NSCLC.^7^, ^9^, ^26^, ^27^, ^28^, ^30^ Moreover, the positive correlation between LIN28B and FGFR1 had further been verified by LIN28B knocked down and FGFR1 inhibited experiments (Figures S3 and S4).

As LIN28 and FGFR1 are strongly related to the progression and prognosis of lung adenocarcinoma patients, and resistance to cisplatin is a major obstacle for the success of NSCLC therapy,^44^ we selected the adenocarcinoma cell line A549 and the cisplatin‐resistant cell line A549R to further validate the inhibitory effects of C1632 on LIN28 and FGFR1. As expected, the qPCR results showed that C1632 decreased LIN28 mRNA levels in both A549 and A549R cells, especially in A549R cells (Figure 2D,E). Our western blot assay showed that C1632 inhibited the expression of FGFR1 as well as FGFR1 phosphorylation and the phosphorylation of the downstream kinase MAPK,^45^ which was abnormally expressed in a series of cancers, and is involved in the regulation of cell proliferation, migration, survival, and apoptosis in a dose‐dependent manner (Figure 2F,G, Figure S5). The fact that C1632 suppresses the expression of LIN28 and blocks FGFR1 signalling supports the idea that it has the potential to serve as an anti‐NSCLC drug (Figure 2D‐G, Figures S3 and S4).

### 3.3 C1632 inhibits the migration of A549 and A549R cells by decreasing the phosphorylation of focal adhesion kinase and the expression of MMP‐9

**FIGURE 3:**
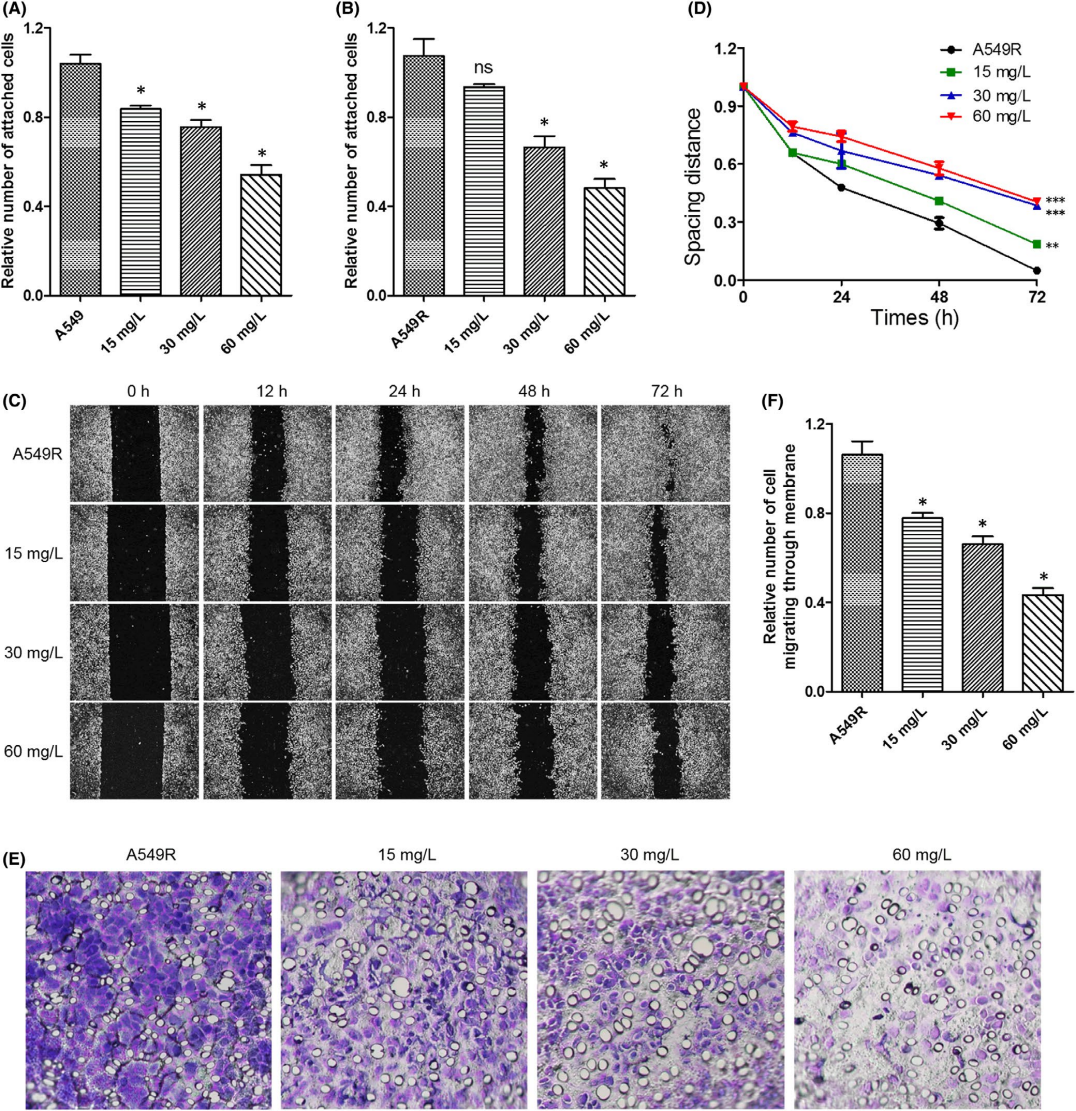
C1632 inhibits the migration and invasion of NSCLC A549 and A549R cells. (A and B) C1632 decreases cell adhesion to extracellular matrix. (C) C1632 inhibits migration of A549R cells in the scratch‐wound healing assay. (D) Quantification of the results in (C). (E) C1632 inhibits migration and invasion of A549R cells in the Transwell assay. (F) Quantification of the results in (E). Values are the average ± SD of three independent experiments. *p* values were calculated using the unpaired Student's *t* test ( ***p < 0.001)

The increase in LIN28 or FGFR1 levels in NSCLC promotes cell migration and proliferation.^23^, ^24^, ^27^ Therefore, we investigated the effects of C1632 on A549 and A549R cell migration. A cell adhesion assay that determines the adhesion between cells and matrix was performed in A549 and A549R cells. As shown in Figure 3A,B, both cell lines displayed a significant decrease in cell adhesion to the matrix after treatment with C1632 in a concentration‐dependent manner.

Cell adhesion is often associated with cell migration.^42^ Therefore, we performed a scratch‐wound healing assay to determine the migration rate of A549 and A549R cells in the presence or absence of C1632. Our results showed that C1632 significantly decreased cancer cell migration (Figure 3C and Figure S6A). After 72 h of treatment, in the control A549R group, 97% of the scratch was covered by migrated A549R cells, while only 60% of the scratch was covered when cells were treated with 60 mg/L C1632 (Figure 3D). Similarly, C1632‐treated A549 cells (60 mg/L) showed a significant decrease in migration rate (Figure S6B). These results demonstrate that C1632 inhibits the migration of cancer cells.

To further confirm the reduced migration ability of cancer cells after C1632 treatment, a Transwell migration and invasion assay was performed (Figure 3E and Figure S6C). The quantitative data demonstrated that A549R and A549 cells treated with C1632 exhibited decreased migration compared with untreated cells (Figure 3F and Figure S6D).

**FIGURE 4:**
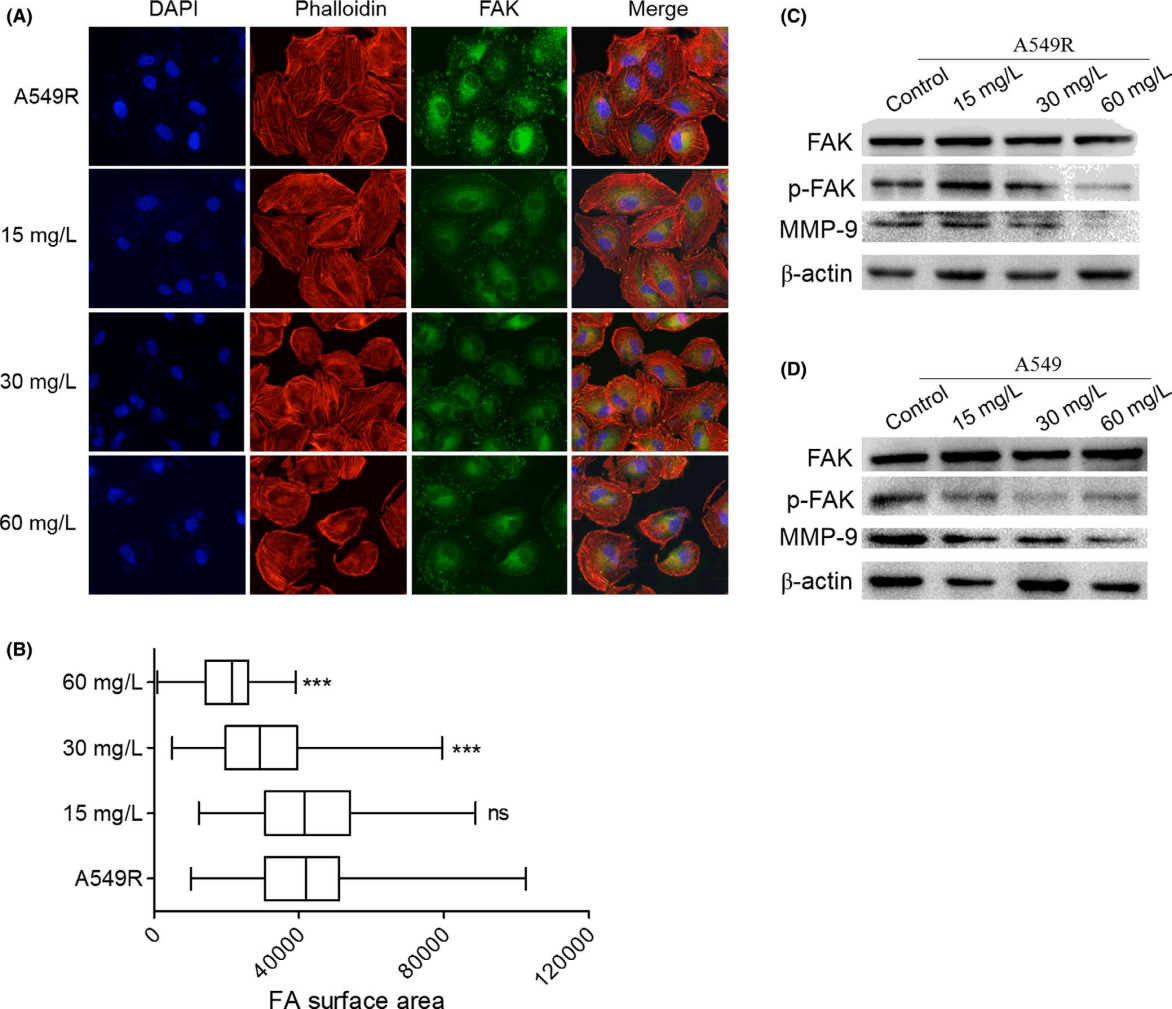
C1632 inhibits the expression and distribution of focal adhesion kinase (FAK) and matrix metalloproteinase 9 (MMP‐9) in NSCLC A549 and A549R cells. (A) Representative images of FAK in C1632‐treated and untreated A549R cells in immunofluorescence assays. Cells were treated with the indicated concentrations of C1632 for 5 days. Cells treated with 0.01% DMSO were selected as a control. Anti‐FAK (blue) and phalloidin (red) were used to visualize FAK and F‐actin, respectively. (B) Focal adhesion surface area, as assessed by FAK and phalloidin staining in C1632‐treated and control A549R cells. Cells were treated with indicated concentrations of C1632 for 5 days. Values are the average ± SD of three independent experiments and ≥500 cells were counted each group. *p* values were calculated using the unpaired Student's *t* test (*p < 0.05, **p < 0.01, ***p < 0.001). (C and D) Western blot determination of FAK, p‐FAK, and MMP‐9 levels in control (0.01% DMSO) and C1632‐treated A549 and A549R cells. Cells were treated with indicated concentrations of C1632 for 5 days and analysed by western blot using antibodies against FAK, p‐FAK, and MMP‐9. β‐actin was used as a loading control. Values are the average ± SD of three independent experiments. *p* values were calculated using the unpaired Student's *t* test (*p < 0.05, **p < 0.01, ***p < 0.001)

Furthermore, we found that C1632 suppressed the phosphorylation of focal adhesion kinase (FAK) and the expression of matrix metalloproteinase‐9 (MMP‐9; Figure 4 and Figure S7), which have been widely implicated in the adhesion, invasion, and migration of cancer cells.^46^ First, IF results showed that C1632 reduced the distribution and the foci formation of FAK in A549R cells in a concentration‐dependent manner (Figure 4A,B, and Figure S7). In addition, western blot results further showed that C1632 efficiently decreased the phosphorylation of FAK and the expression of MMP‐9 (Figure 4C,D). Taken together, these findings support the conclusion that C1632 inhibited the migration of A549 and A549R cells via suppressing the phosphorylation of FAK and the expression of MMP‐9, which might be a result from the reduction in LIN28 expression and blockage of FGFR1 signalling.

### 3.4 C1632 suppresses the colony formation of A549 and A549R cells by inhibiting DNA replication and inducing G0/G1 cell cycle arrest

**FIGURE 5:**
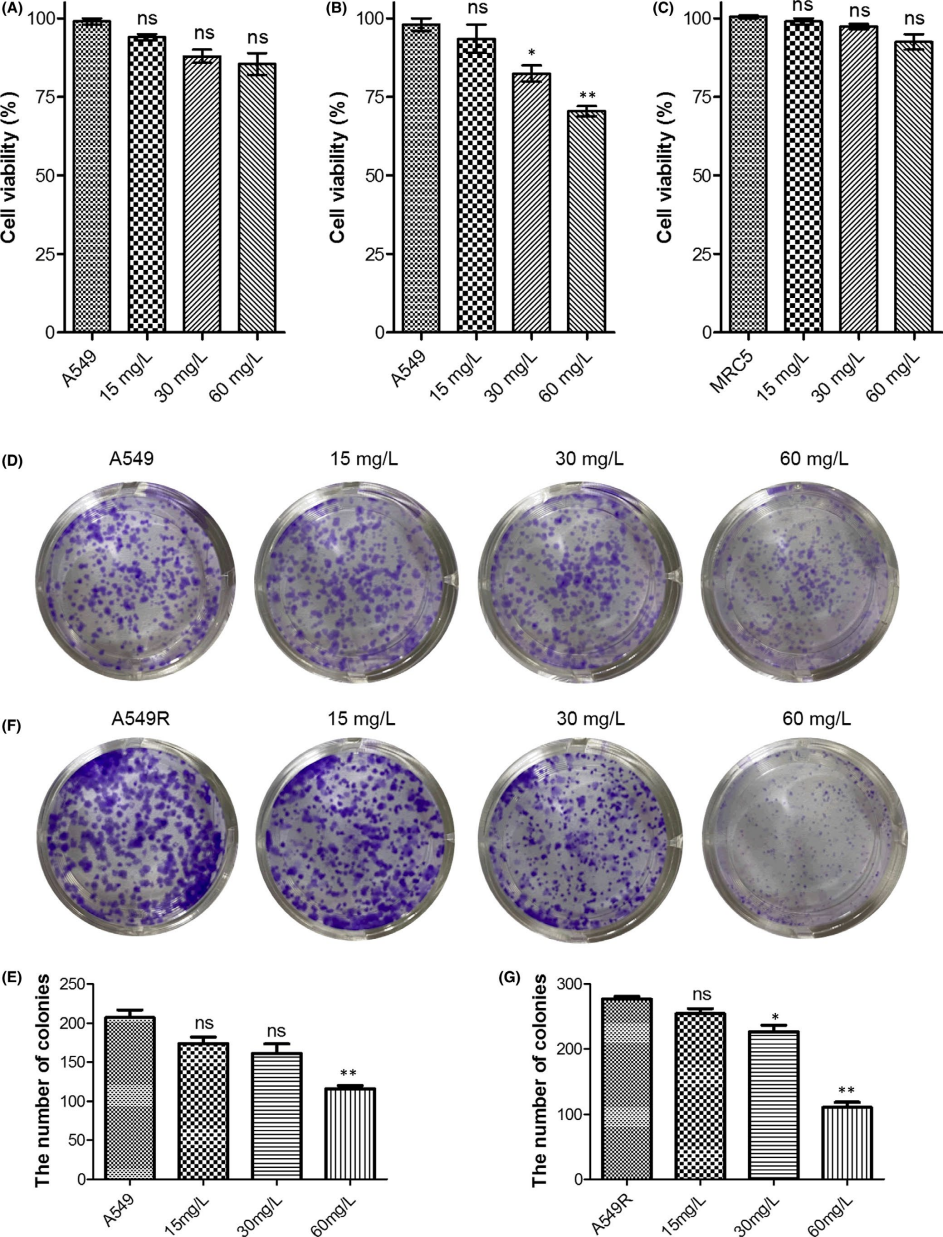
C1632 inhibits cell viability and suppresses the colony formation of A549 and A549R cells. Viability of A549 (A), A549R (B), and MRC5 (C) cells was measured after C1632 treatment for 5 days by MTT assay. (D) Representative light microscopy images of crystal violet‐stained colonies of C1632‐treated A549 cells. Cells were treated with 0.01% DMSO or indicated concentrations of C1632 for 5 days prior to the colony formation assay. (F) The same as in D for A549R cells. (E and G) Quantification of data in (D) and (F), respectively. Values are the average ± SD of three independent experiments. *p* values were calculated using the unpaired Student's *t* test (*p < 0.05, **p < 0.01)

C1632 decreased the viability of A549 and A549R cells at a high dose (60 mg/L), but not at a low dose (15 mg/L; Figure 5A,B). It is worthy to note that a high dose (60 mg/L) of C1632 almost had no cytotoxicity on the human normal foetal lung fibroblast cell line MRC5 (Figure 5C). Consistent with the MTT assay, the colony formation assay showed that C1632 inhibited the formation of colony units in a dose‐dependent manner, and the inhibition of colony formation was stronger in A549R cells than in A549 cells, in agreement with the cytotoxicity on A549 and A549R cells (Figure 5D,F). The annexin V/PI apoptotic assay and the SA‐β gal staining assay revealed that C1632 treatment, even at a high dose, did not significantly increase apoptosis or senescence (Figures S8 and S9), suggesting that the inhibitory effects of C1632 on colony formation are not due to cytotoxicity‐induced cell death.

**FIGURE 6:**
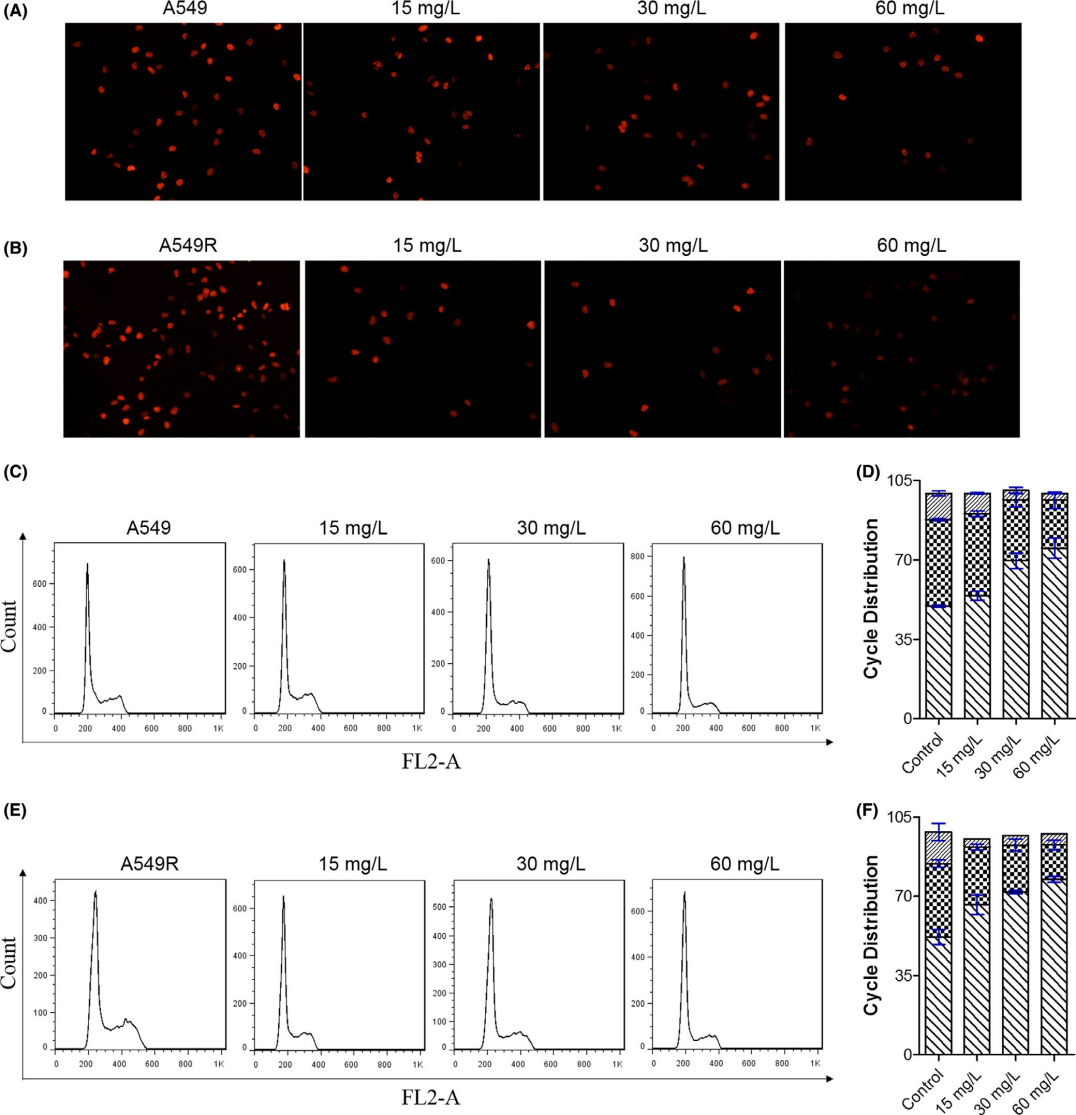
C1632 inhibits DNA replication and induces G0/G1 cell cycle arrest of A549 and A549R cells. (A) Representative images of C1632‐treated and untreated A549 cells in Edu staining assays. Cells were treated with the indicated concentrations of C1632 for 5 days. Cells treated with 0.01% DMSO were selected as a control. (B) The same as in A for A549R cells. (C) Representative images of C1632‐treated A549 cells in FACS analysis. Cells were treated with the indicated concentrations of C1632 for 5 days. Cells treated with 0.01% DMSO were selected as a control. (D) Quantification of the results in (C). (E) The same as in (C) for A549R cells. (F) Quantification of the results in E. Values are the average ± SD of three independent experiments

Additionally, an Edu staining assay and flow cytometry were performed to further investigate whether C1632 inhibited the colony formation of A549 and/or A549R cells by DNA replication inhibition and cell cycle arrest. The results showed that C1632 treatment led to a significant inhibition of DNA replication in A549 and A549R cells in a dose‐dependent manner (Figure 6A,B). Consequently, C1632 treatment arrested A549 and A549R cells at the G0/G1 phase, reducing the percentage of cells in both the S and the G2/M phase, in a dose‐dependent manner (Figure 6C–F). In conclusion, C1632 inhibited cell viability and colony formation by suppressing DNA replication and induced cell cycle arrest in the G0/G1 phase, slowing the transition to the S phase.

### 3.5 C1632 suppresses the growth of A549R xenograft tumours in mice

**FIGURE 7:**
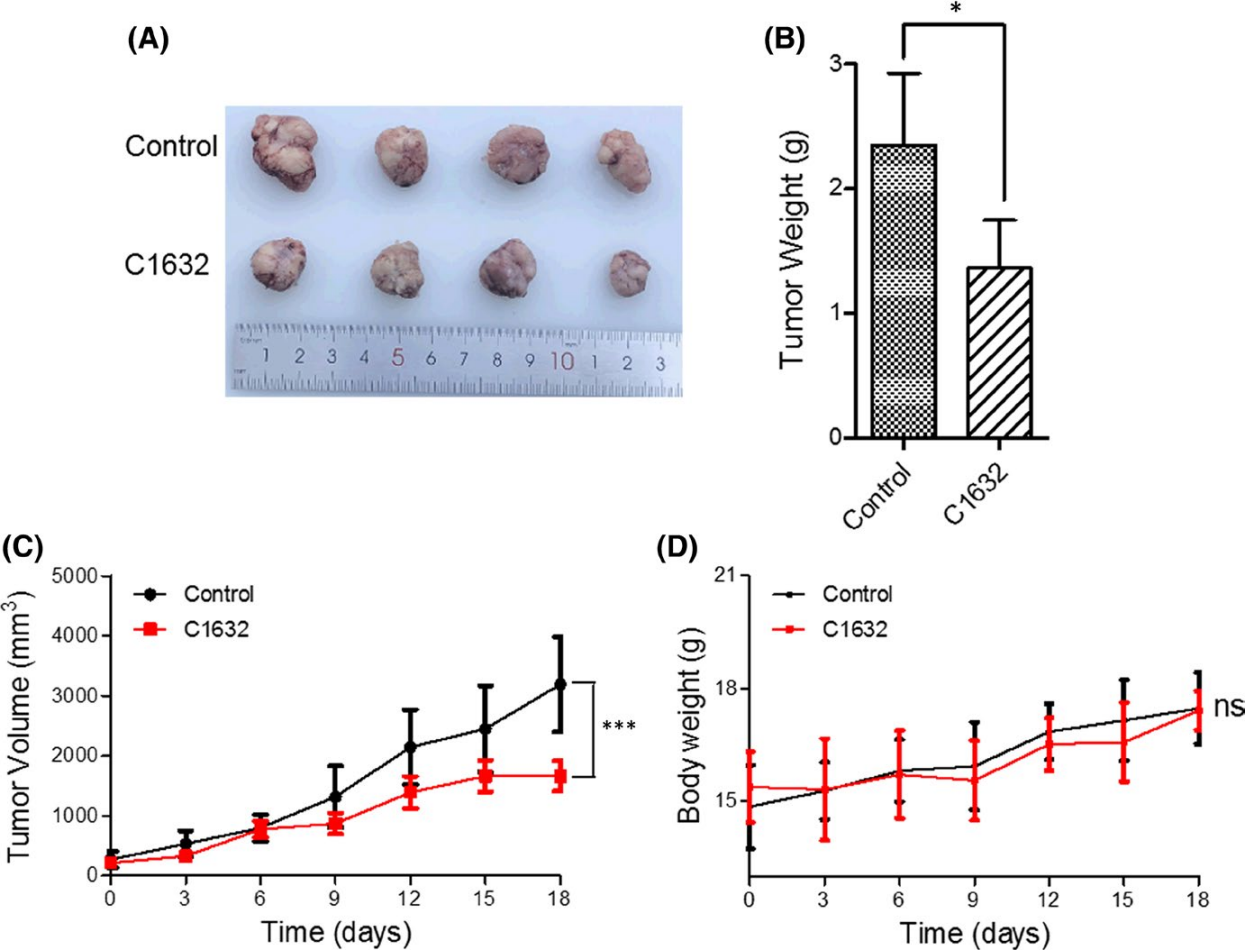
C1632 suppresses the growth of A549R xenograft tumours in mice. (A) Female 4‐week‐old mice were injected (i.p.) with inoculum containing 1 × 10^6^ A549R cells; 30 mg/kg C1632 dissolved in phosphate‐buffered saline (PBS) was i.v. injected into the tail vein every 2 days for 18 days. In the control group, the same volume of PBS was injected. Representative images of xenograft tumours from treated and untreated mice are shown (*n* = 4 per group). (B) Quantification of data in (A). (C) The tumour volume of the mice was measured over the treatment period. (D) The body weight of the mice was measured over the treatment period. *p* values were calculated using the unpaired Student's *t* test (*p < 0.05, ***p < 0.001)

The above results prompted us to examine the endogenous antitumour activity of C1632 on A549R xenograft tumours in mice. Two weeks after injection with the cancer cell inoculum, and then every 2 days thereafter, mice were injected in the caudal vein with 30 mg/kg C1632. Although tumours were still visible after 18 days in the treated group, the tumour size was smaller than in the untreated group (untreated, mean ± SD = 2.35 ± 0.43 g; treated, mean ± SD = 1.36 ± 0.27 g; *p* < 0.05) (Figure 7A,B). In addition, C1632 suppressed the growth of xenograft tumour cells in a time‐dependent manner (Figure 7C). Treatment did not affect the body weight of mice inoculated with A549R cells (Figure 7D). These results indicate that C1632 inhibits the growth of A549R xenograft tumours in mice and had no the toxi‐side effects on the body.

## 4 DISCUSSION

It is now recognized that tumour drug distribution and bioavailability are important factors for effective tumour treatment.^47^, ^48^ Our results demonstrated that C1632 mainly accumulated in the lung after oral administration, with up to 44.45% bioavailability and limited inhibitory effects on CYP450 isoenzymes (Figure 1). Our results also showed that C1632 reduced the cell viability of NSCLC A549 and A549R cells, while it almost had no toxicity to MRC5 cells in vitro (Figure 5A‐C). These observations strongly suggest that C1632 possesses a great therapeutic potential in lung cancer, especially NSCLC, which accounts for 85% of lung cancer cases.

Previous studies showed that either LIN28 or FGFR1 is strongly correlated with the progression of NSCLC,^22^, ^27^ and FGFR1 inhibitors achieved a definite therapeutic effect of NSCLC in the clinic and in an animal model.^30^, ^31^, ^34^ However, FGFR1‐targeted therapies are susceptible to drug resistance,^1^, ^26^, ^28^ and there is still no LIN28 inhibitor available for NSCLC treatment. In this study, we demonstrated that it had a positive correlation between FGFR1 and LIN28B (Figures S3 and S4), and C1632 suppressed the expression of LIN28 and blocked FGFR1 signalling in NSCLC A549 and A549R cells (Figure 2), resulting in an inhibition of migration (Figure 3 and Figure S6) and proliferation (Figure 5) of A549 and A549R cells in vitro and in vivo.

Fibroblast growth factor receptor 1 is involved in the regulation of FAK phosphorylation and MMP‐9 expression via the FGFR‐extracellular regulated kinase‐FAK pathway,^49^ which has been implicated in the invasion, metastasis, and motility of cancer cells.^46^ The results presented here showed that C1632 blocked the FGFR1 signalling pathway by inhibiting the phosphorylation of FGFR1 (Figure 2F,G), and consequently significantly inhibited the phosphorylation of FAK and the expression of MMP‐9 (Figure 4C,D). Additionally, LIN28 has also been reported to promote cancer cell metastasis.^19^ Thus, the anti‐migration effects of C1632 (Figure 3 and Figure S7) might be the result of the suppression of LIN28 expression and the simultaneous blockage of the FGFR1 signalling pathway (Figure 2D‐G). Besides metastasis, LIN28 and FGFR1 are closely correlated with cancer cell growth and drug resistance.^23^, ^27^, ^44^ Consistent with these previous reports, our results also demonstrated the inhibitory effects of C1632 on the viability and colony formation ability of NSCLC A549 and A549R cells in vitro and in vivo (Figures 5 and 7). Our results also reveal that C1632 treatment inhibited DNA replication of NSCLC A549 and A549R cells and induced G0/G1 cell cycle arrest (Figure 6), indicating that the anti‐NSCLC effect of C1632 is not only due to increased cell death (Figures S8 and S9). Interestingly, compared with A549 cells, C1632 exerts the same or even stronger anti‐migration and anti‐proliferation effects on A549R cells, regardless of drug resistance (Figures 3 and 4 and Figure S6).

Collectively, these results revealed that C1632 simultaneously suppressed LIN28 expression and blocked FGFR1 signalling and that C1632 is able to rapidly inhibit migration and proliferation of NSCLC cells, regardless of drug resistance, in vivo, indicating that it has the potential to act as an anticancer agent for NSCLC treatment.

## CONFLICT OF INTERESTS

The authors confirm that there are no conflicts of interest.

## AUTHOR CONTRIBUTIONS

**Jing‐yi Chen:** Investigation (equal); Project administration (equal); Writing – original draft (equal). **Yu‐jing Chen:** Data curation (equal); Investigation (equal); Writing – original draft (equal). **Lu Liu:** Formal analysis (equal); Investigation (equal); Validation (equal). **Xiang‐xiang Jin:** Data curation (equal); Formal analysis (equal). **Zhe Shen:** Investigation (equal); Methodology (equal). **Wen‐bin Chen:** Data curation (equal); Methodology (equal). **Teng Yang:** Investigation (equal). **Si‐bei Xu:** Investigation (equal). **Guang‐bao Wang:** Methodology (equal). **Yi‐nuo Cheng:** Resources (equal); Software (equal). **De‐zhi Cheng:** Conceptualization (equal); Writing – original draft (equal); Writing – review & editing (equal). **Zhi‐guo Liu:** Supervision (equal); Visualization (equal); Writing – original draft (equal). **Xiao‐hui Zheng:** Conceptualization (lead); Funding acquisition (lead); Project administration (equal); Supervision (lead); Writing – original draft (equal); Writing – review & editing (equal).

## Supporting information

Appendix S1

## Data Availability

All data in this study are available if requested.
