# Supplementary material for: C1632 suppresses the migration and proliferation of non‐small‐cell lung cancer cells involving LIN28 and FGFR1 pathway
Source: J Cell Mol Med. 2021 Dec 16;26(2):422–35. doi: 10.1111/jcmm.17094 (PMC8743659; doi:10.1111/jcmm.17094)
Supplement: Supplementary file 1 — Appendix S1 [file JCMM-26-422-s001.docx]

**C1632 suppresses the migration and proliferation of non-small cell lung cancer cells** **involving LIN28 and FGFR1 pathway**

Jing-yi Chen^1,#^, Yu-jing Chen^1,#^, Lu liu^1,#^, Xiang-xiang Jin^1^, Zhe Shen^1^, Wen-bin Chen^1^, Teng Yang^1^, Si-bei Xu^1^, Guang-bao Wang^1^, Yi-nuo Cheng^1^, De-zhi Chen^2,*^, Zhi-guo Liu^1,*^, Xiao-hui Zheng^1,*^

^1^ Chemical Biology Research Center at School of Pharmaceutical Sciences, Wenzhou Medical University, 1210 University Town, Wenzhou, Zhejiang 325035, China

^2^ Department of Thoracic Surgery, The First Affiliated Hospital of Wenzhou Medical University, Wenzhou 325000, Zhejiang, People’s Republic of China

^#^ These authors contribute equally to this work.

* Corresponding author:

De-zhi Cheng, Ph.D, Director

Department of Thoracic Surgery, The First Affiliated Hospital of Wenzhou Medical University, Wenzhou 325000, Zhejiang, People’s Republic of China

Tel: (+86)-577- 55578020; Fax: (+86) -577- 55578020

E-mail: dezhicheng@sina.com

Zhi-guo Liu, Ph.D, Professor

Chemical Biology Research Center at School of Pharmaceutical Sciences, Wenzhou Medical University. 1210 University Town, Wenzhou, Zhejiang 325035, China

Tel: (+86)-577-86699892; Fax: (+86)-577-86699892

E-mail: lzgcnu@163.com

And Xiao-hui Zheng, Ph.D, Associate Professor

Chemical Biology Research Center at School of Pharmaceutical Sciences, Wenzhou Medical University. 1210 University Town, Wenzhou, Zhejiang 325035, China

Tel: (+86)-577-86699892; Fax: (+86)-577-86699892

E-mail: zhengxh@wmu.edu.cn

**Supplementary Table 1. Pharmacokinetic parameters of C1632 administrated through oral and intravenous.**

| **PK parameters** | ***p.o.* 20 mg/kg** | ***i.v.* 4 mg/kg** |
| --- | --- | --- |
| AUC(0-t) (ug/L*h) | 9098.60±3828.51 | 4093.81±1632.66 |
| MRT(0-t) (h) | 1.88±0.42 | 1.57±0.74 |
| t1/2z (h) | 1.05±0.60 | 1.14±0.52 |
| Tmax (h) | 1.04±0.78 | 0.53±0.74 |
| Vz/F (L/kg) | 4.54±4.68 | 2.05±1.66 |
| CLz/F (L/h/kg) | 2.77±1.74 | 1.13±0.51 |
| Cmax (ug/L) | 4238.18±2683.45 | 2066.67±338.23 |
| F | 44.45% | |


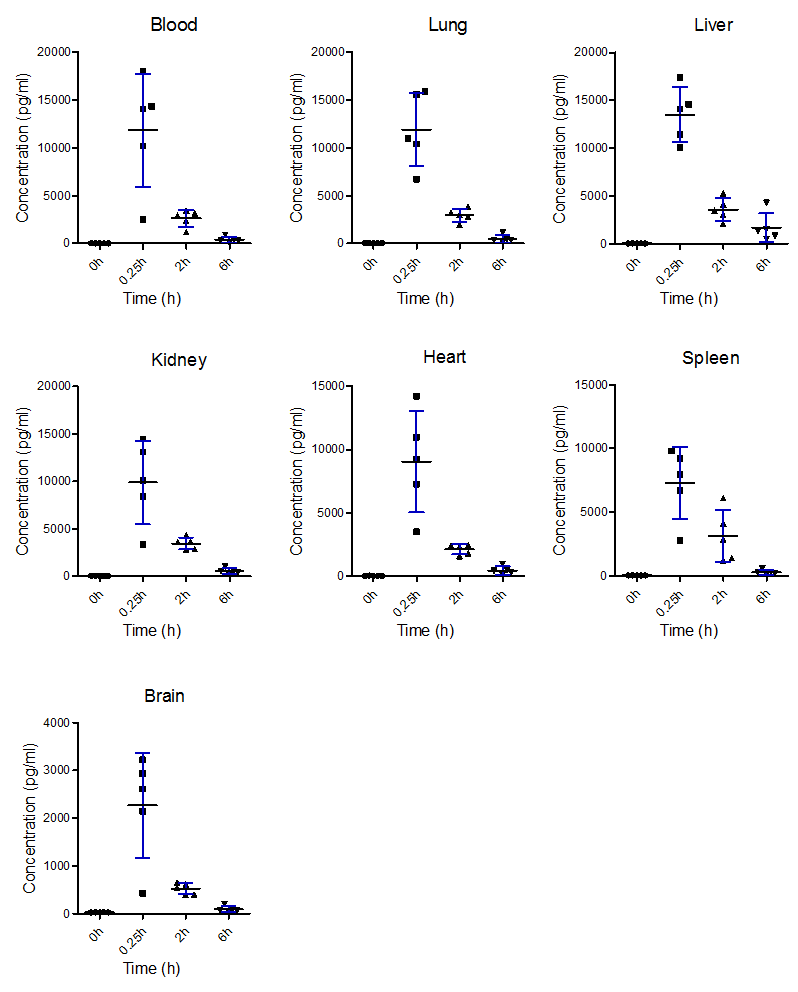


**Supplementary Figure 1.** Mean concentration of C1632 in tissues at different time (0.25, 2 and 6 h) after tail vein administration of 20 mg/kg C1632 in mouse (n=6).


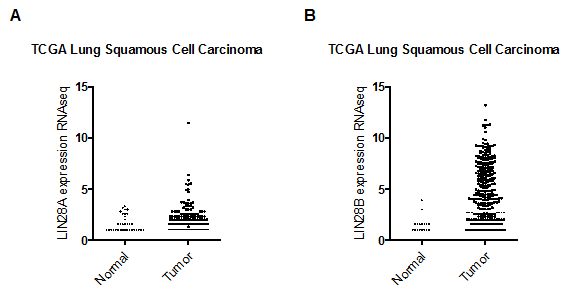


**Supplementary Figure 2.** **The over expression of LIN28 in lung squamous cell cancer detected by transcriptome analysis of TCGA lung squamous cell cancer samples.** **A**, Plots were based on the expression values of LIN28A in lung squamous cell cancer samples compared to normal tissues (n= 575). **B**, the same as A, except LIN28B was used (n= 524).

**Supplementary Figure 3. LIN28 knockdown inhibited the expression of FGFR1 and the protein phosphorylation. A** and **B**, qRT-PCR showed the abundance of LIN28B mRNA in A549 (A) and A549R (B) cancer cell lines (NC) and A549/A549R cells transfected with siRNA (siRNA_1 and siRNA_2). **C**, Western blot assay evaluated the total protein of LIN28B and FGFR1, as well as the phosphorylation of FGFR1 in A549 cells. **D**, The same as in C for A549R cells.

**Supplementary Figure 4. Ponatinib inhibited FGFR1, suppressing the expression of LIN28B in A549 (A) and A549R (B) cell lines.**

**Supplementary Figure 5. C1632 inhibited the MAPK expression and phosphorylation in NSCLC A549 and A549R cells.** **A**, C1632 inhibited MAPK expression and phosphorylation in A549 cells. A549 cells were treated with C1632 indicated concentrations for 5 days before cell lysis. Western blot analysis was used to determine the protein expression of p-MAPK, and MAPK. GAPDH was used as loading control. The results shown are representative of three replicated experiments. **B**, The same as in A for A549R cells.





**Supplementary Figure 6. C1632 inhibits the migration and invasion of NSCLC A549 cells.** **A**, C1632 inhibits A549 cells migration in scratch-wound healing assay. **B**, Quantification of A. **C**, C1632 inhibits A549 cells migration and invasion in a transwell assay. **D**, Quantification of C. Values are the average ± SD of three independent experiments. *p* values were calculated using the unpaired student’s *t*-test (**p*< 0.05, ***p*< 0.01, ****p*<0.001).


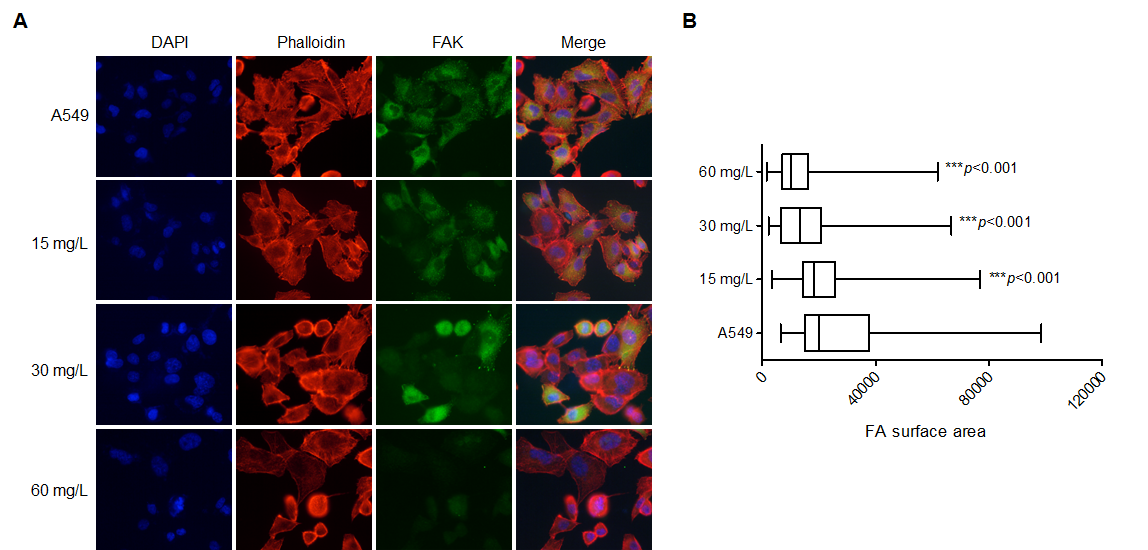


**Supplementary Figure 7. C1632 inhibits the expression and distribution of focal adhesion kinase (FAK) in NSCLC A549 cells. A**, Representative images of focal adhesion kinase (FAK) in C1632-treated and untreated A549 cells in immunofluorescence assays. Cells were treated with the indicated concentration of C1632 (15, 30 or 60 mg/L) for 5 days. Cells treated with 0.01% DMSO were selected as a control. Antibody to FAK (blue) and phalloidin (red) were used to visualize FAK and F-actin, respectively. **B**, Focal adhesion surface area assessed through FAK and phalloidin staining in C1632-treated and control A549 cells. Cells were treated with indicated concentration of C1632 (15, 30 or 60 mg/L) for 5 days. Values are average ± SD of at least three independent experiments and ≥500 cells were counted each group. The statistical significance was calculated using the unpaired student’s two-tailed *t*-test with the *p*-values (**p* < 0.05, ***p* < 0.01, ****p* <0.001).


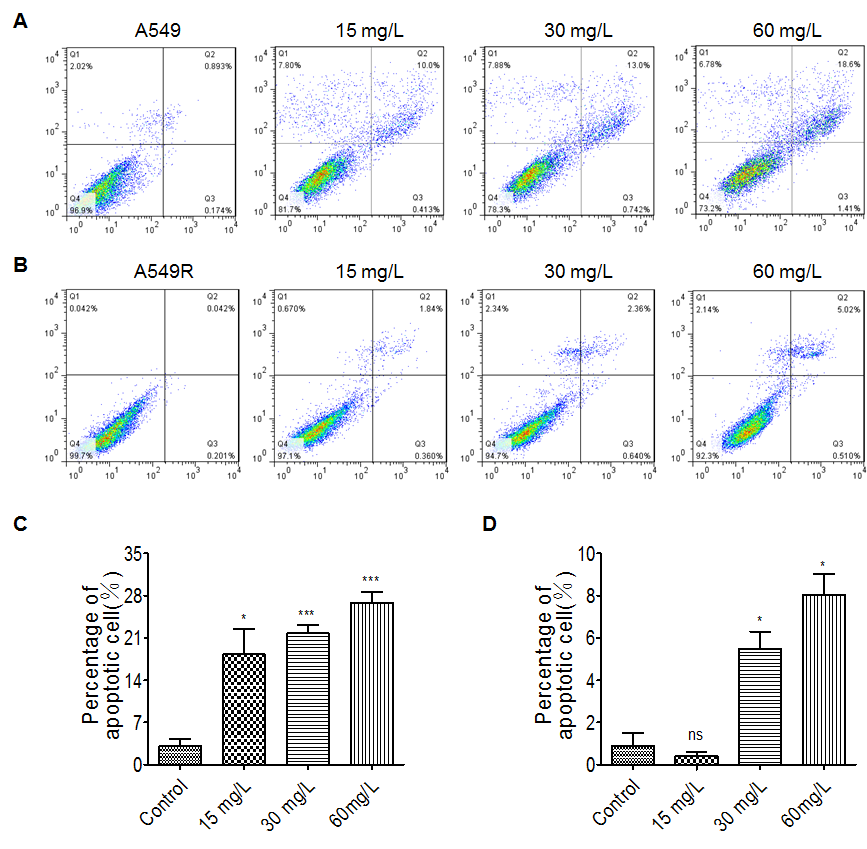


**Supplementary Figure 8. C1632 treatment induces apoptosis of NSCLC A549 cells, while had limited effects on A549R cells.** FACS analysis was performed to detect the apoptotic A549 cells (**A**) and A549R cells (**B**) treated with the indicated concentration of C1632 (15, 30 or 60 mg/L) for 5 days. Cells treated with 0.01% DMSO were selected as a control. **C** and **D**, Quantification of A and B. Values are average ± SD of three independent experiments. Unpaired Student’s two-tailed *t* test was used to determine the statistical significance (**p*< 0.05, ***p*< 0.01, ****p*<0.001).


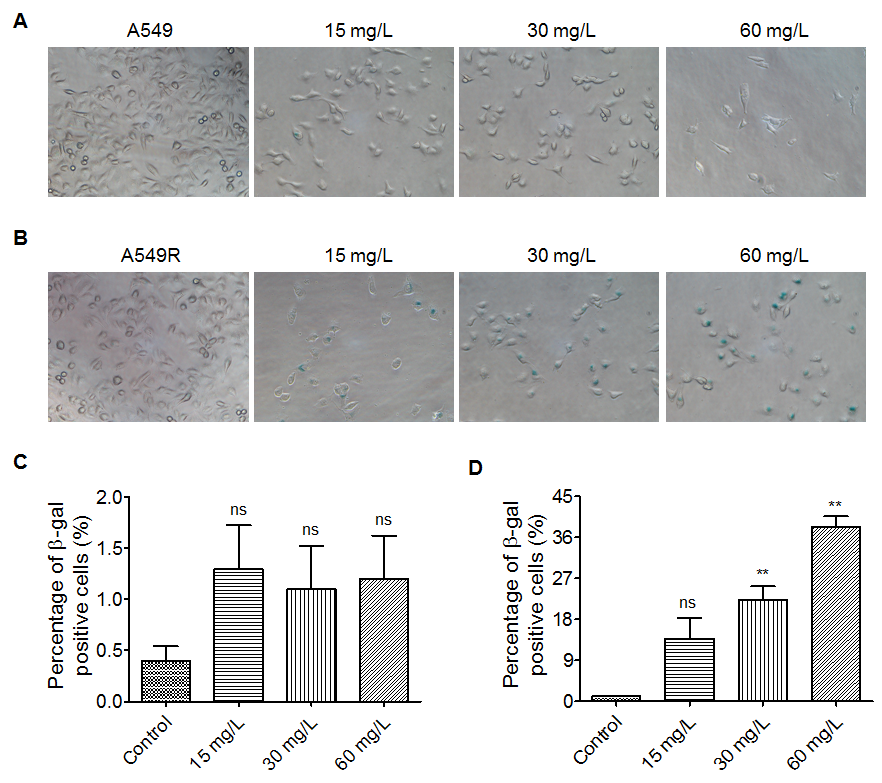


**Supplementary Figure 9. C1632 treatment (15, 30 and 60 mg/L) did not induce the senescence of NSCLC A549 cells, but induce the senescence of A549R cells. A**, Representative results of β-gal staining assay of C1632 treated A549 cells at the indicated concentration of 5 days. **B**, the same as A, except A549R cells were used. **C**, Quantitation of the percentage of senescent (β-gal positive) cells in A. **D**, Quantitation of the percentage of senescent (β-gal positive) cells in B. Values are average ± SD of at least three independent experiments and ≥500 cells were counted each group. The statistical significance was calculated using the unpaired student’s two-tailed *t*-test with the p-values (**p* < 0.05, ***p* < 0.01, ****p* <0.001).
